# Memory-efficient RNA energy landscape exploration

**DOI:** 10.1093/bioinformatics/btu337

**Published:** 2014-05-14

**Authors:** Martin Mann, Marcel Kucharík, Christoph Flamm, Michael T. Wolfinger

**Affiliations:** ^1^Bioinformatics Group, Department of Computer Science, University of Freiburg, 79110 Freiburg, Germany, ^2^Institute for Theoretical Chemistry, University of Vienna, 1090 Vienna, ^3^Center for Integrative Bioinformatics Vienna, Max F. Perutz Laboratories, University of Vienna, Medical University of Vienna, and ^4^Department of Biochemistry and Molecular Cell Biology, Max F. Perutz Laboratories, University of Vienna, A-1030 Vienna, Austria

## Abstract

**Motivation:** Energy landscapes provide a valuable means for studying the folding dynamics of short RNA molecules in detail by modeling all possible structures and their transitions. Higher abstraction levels based on a macro-state decomposition of the landscape enable the study of larger systems; however, they are still restricted by huge memory requirements of exact approaches.

**Results:** We present a highly parallelizable local enumeration scheme that enables the computation of exact macro-state transition models with highly reduced memory requirements. The approach is evaluated on RNA secondary structure landscapes using a gradient basin definition for macro-states. Furthermore, we demonstrate the need for exact transition models by comparing two barrier-based approaches, and perform a detailed investigation of gradient basins in RNA energy landscapes.

**Availability and implementation:** Source code is part of the C++ Energy Landscape Library available at http://www.bioinf.uni-freiburg.de/Software/.

**Contact:** mmann@informatik.uni-freiburg.de

**Supplementary information:** Supplementary data are available at *Bioinformatics* online.

## 1 INTRODUCTION

The driving force of disordered systems in physics, chemistry and biology is characterized by coupling and competing interaction of microscopic components. At a qualitative level, this is reflected by the potential energy function and often results in complex topological properties induced by individual conformational degrees of freedom. It seems fair to say that it is practically impossible to compute dynamic and thermodynamic properties directly from the Hamiltonian of such a complex system. However, analyzing the underlying energy landscape and its features directly provides a valuable alternative.

Here, we focus on RNA molecules and their folding kinetics. RNAs are key players in cells acting as regulators, messengers, enzymes and many more roles. In many cases, a specific structure is crucial for biological specificity and functionality. The formation of these functional structures, i.e. the folding process, can be studied at the level of RNA energy landscapes (Flamm and Hofacker, 2008; Geis *et al.*, 2008).

RNA is composed of the biophysical alphabet {A,C,G,U} and has the ability to fold back onto itself by formation of discrete base pairs, thus forming secondary structures. The latter provide a natural coarse-graining for the description of the thermodynamic and kinetic properties of RNA because, in contrast to proteins, the secondary structure of RNA captures most of the folding free energy. This is accommodated by novel approaches for predicting 3D RNA structures from secondary structures (Popenda *et al.*, 2012).

Formally, an RNA secondary structure is defined as a set of base pairs between the nuclear bases complying with the rules: (i) only A-U, G-C and G-U pairings are allowed, (ii) any base is involved in maximal one base pair and (iii) the structure is nested, i.e. there are no two base pairs with indices (*i*, *j*), (*k*,*l*) with *i* < *k* < *j* < *l*. Summation over the individual base pair binding energies and entropic contributions for unpaired bases defines the energy function *E* (Freier *et al.*, 1986; Hofacker *et al.*, 1994; Tinoco *et al.*, 1971). The degeneracy of this energy definition is countered via a structure ordering based on their string encoding (Flamm *et al.*, 2002). We refer to the literature (Chen, 2008; Flamm and Hofacker, 2008) for more details.

In this work, we study the folding kinetics of RNA molecules by means of a discrete energy landscape approach. While stochastic folding simulations based on solving the Master equation are limited to relatively short sequence lengths (Aviram *et al.*, 2012; Flamm *et al.*, 2000b), a common approach to studying biopolymer folding dynamics is using a coarse-grained model that partitions the energy landscape into distinct basins of attraction, thus assigning macro-states to each basin (Wolfinger *et al.*, 2004). The basin decomposition and computation has been described in different contexts, including potential energy landscapes (Heuer, 2008), RNA kinetics (Flamm and Hofacker, 2008) and lattice protein folding (Tang and Zhou, 2012; Wolfinger *et al.*, 2006). Given appropriate transition rates between macro-states (optionally composed of rates among micro-states that form a macro-state), the dynamics can be modeled as continuous-time Markov process and solved directly by numerical integration (Wolfinger *et al.*, 2004). While suitable for system sizes up to ∼10 000 states, improvements to this approach are currently subject to our research, allowing investigation of up to a few hundred thousand states by incorporating sparsity information and additional approximations.

The crucial step in the procedure sketched above is to obtain the transition rates between macro-states. Global methods for complete (Flamm *et al.*, 2002) or partial (Kubota and Hagiya, 2005; Sibani *et al.*, 1999; Wolfinger *et al.*, 2006) enumeration of the energy landscape are not applicable to large systems because of memory restrictions. On the other side, sampling with high precision requires long sampling times (Mann and Klemm, 2011). Therefore, approximating the energy landscape by a subset of important local minima, gained via sampling approaches or spectroscopic methods (Alemán *et al.*, 2008; Fürtig *et al.*, 2007; Rinnenthal *et al.*, 2011), and transition paths between them (Noé and Fischer, 2008) has been investigated in the past (Kucharík *et al.*, 2014; Tang *et al.*, 2005, 2008).

We propose a novel, highly parallelizable and memory-efficient local enumeration approach for computing exact transition probabilities. While the method is intrinsically generic and can be readily applied to other discrete systems, we exemplify the concept in the context of energy landscapes of RNA secondary structures, based on the Turner energy model (Xia *et al.*, 1998), as implemented in the Vienna RNA Package (Hofacker *et al.*, 1994; Lorenz *et al.*, 2011) and the Energy Landscape Library (ELL; Mann *et al.*, 2007). We evaluate the memory efficiency and dynamics quality for different RNA molecules and report features of gradient basin macro-states in RNA energy landscapes.

## 2 DISCRETE ENERGY LANDSCAPES

In the following, we will define energy landscapes for two levels of abstraction: the *microscopic level* covers all possible (micro-) states of a system and its dynamics, whereas the *macroscopic level* enables a more coarse-grained model of the system’s dynamics, based on a partitioning of all micro-states into macro-states. The macroscopic view is required when studying the dynamics of larger systems.

### 2.1 Microscopic level

Discrete energy landscapes are defined by a triple (X,E,M) given a finite set of (micro-)states *X*, an appropriate energy function E:X→ℝ, and a symmetric neighborhood relation M:X→P(X) (also known as move set), where P(X) is the power set of *X*. The neighborhood M(x) is the set of all neighboring states that can be directly reached from state *x* by a simple move set operation.

Consequently, RNA energy (folding) landscapes can be defined at the level of secondary structures, which represent the micro-states x∈X. An RNA structure *y* is neighbored to a structure *x* (y∈M(x)), if they differ in one base pair only. Although alternative move set definitions are possible (Flamm et al., 2000b), they are not considered in this work for simplicity.

Within this work, we consider time-discrete stochastic dynamics based on Metropolis transition probabilities *P* at inverse temperature β:

(1) $$\begin{array}{l} p_{x\to y}=\Delta^{-1}\min\{\exp(-\beta [E(y)-E(x)]),1\}\\ =\Delta^{-1}\min\{w(y)/w(x),1\} \end{array}$$

(2) withw(x)=exp(−βE(x))

(3) $$\text{and}\;\Delta =\underset{x\in X}{\max}|M(x)|.$$

*w*(*x*) is the Boltzmann weight of *x*. Normalization is performed via the constant Δ, which is the maximally possible number of neighbors/transitions of any state. The transition probability $p_{x\to y}$ is only defined for neighboring states, i.e. y∈M(x).

### 2.2 Macroscopic level

Although desirable, studying dynamic properties at the microscopic level is often not feasible because of the vastness of the state space *X*, even for relatively small systems. An alternative approach is coarse graining, i.e*.* lumping many micro-states into fewer macro-states, such that the microscopic dynamics is resembled as closely as possible (Wolfinger *et al.*, 2004).

This can be achieved by partitioning of the state space *X* with a mapping function F:X→B that uniquely assigns any micro-state in *X* to a macro-state in *B*. With $F^{-1}(b)$ we denote the inverse function that gives the set of all *F*-assigned states for a macro-state b∈B. Following (Flamm and Hofacker, 2008; Kramers, 1940; Mann and Klemm, 2011; Wolfinger *et al.*, 2004), we will use the simplifying assumption that the probability of the system to be in micro-state *x* while it is in macro-state b∈B is given by

(4) $$P_{b}(x)\;=\{\begin{array}{cc} w(x)Z_{b}^{-1} & \text{if}\;x\in F^{-1}(b)\\ 0 & \text{otherwise} \end{array}$$

(5) $$\;\text{with}\;Z_{b}\;=\sum_{y\in F^{-1}(b)}w(y).$$

Based on this, we can define the macroscopic transition probabilities $q_{b\to c}$ between macro-states b,c∈B by means of the microscopic probabilities *P* from Equation (1) as follows:
(6)
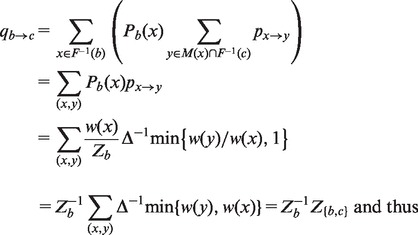

(7)
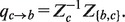



Equation (6) considers all microscopic transitions x→y from a micro-state *x* in *b* to a micro-state *y* in *c*, based on the probability of *x* $\left(P_{b}(x)\right)$ and the transition probability $p_{x\to y}$. The energetically higher micro-state of each such transition contributes to the partition function of all transition states between *b* and *c*, $Z_{\left\{b,c\right\}}$ [Equations (6 and 7)]. Consequently, $Z_{\left\{b,c\right\}}\equiv Z_{\left\{c,b\right\}}$, i.e. the transition state partition function is direction-independent.

Within this work, we use the common gradient basin partitioning of *X* following (Doye, 2002; Flamm *et al.*, 2002; Flamm and Hofacker, 2008; Mann and Klemm, 2011). A gradient basin is defined as the set of all states who have a steepest descent (gradient) walk ending in the same local minimum, where *x̌* is a local minimum if ∀_*y*∈*M*(*x̌*)_ : *E*(*x̌*) < *E*(*y*). In this context, the set of macro-states *B* is given by the set of all local minima of the landscape, whose number is drastically smaller than that of all micro-states (Lorenz and Clote, 2011). The mapping function *F*(*x*) applies a gradient walk starting in *x*, thus assigning it a local minimum *x̌* and a macro-state *b*. Here, the minimum is used as a representative for the macro-state composed of the gradient basin.

A coarse abstraction of the macro-state transition probabilities can be obtained by an Arrhenius-like transition model (Wolfinger *et al.*, 2004). Here, the transition probability is dominated by the minimal energy barrier that needs to be traversed to go from one state to another. Formally, given two states *x* and *y*, one has to identify the path $p=(x_{1},\ldots ,x_{l})\in X^{l},l>1$ with $x_{1}=x$, $x_{l}=y$ and $\forall i<l:x_{i+1}\in M(x_{i})$, with lowest energy maximum. Arrhenius barrier-based transition probabilities are thus defined by

(8) $$a_{x\to y}=A\;\exp(-\beta (E(x,y)-E(x)))\;\text{with}$$

(9) $$E(x,y)=\underset{p\in X^{*}}{\min}\underset{\underset{i}{x}\in p}{\max}(E(x_{i}))$$

where *A* is an intrinsically unknown pre-exponential factor. For macro-state transitions based on a gradient basin partitioning, transition probabilities can be approximated by Arrhenius probabilities among local minima of macro-states. In this context, it is important to note that this transition model does not enforce neighborhood of the macro-states. The impact on modeling quality of such an Arrhenius-based model is evaluated in Section 4. We will now present approaches for the exact determination of the macro-state transition probabilities for a given landscape and partitioning.

## 3 MACRO-STATE TRANSITION PROBABILITIES

Following the rationale presented above, all macroscopic transition rates need to be determined to study the coarse-grained dynamics. Given Equation (6), the partition function *Z_b_* [Equation (5)] and adjunct partition functions of transition states $Z_{\left\{b,c\right\}}$ to adjacent c≠b have to be computed for each macro-state *b*.

A direct approach is brute-force enumeration of *X*, computing *F*(*x*) for each micro-state x∈X and updating $Z_{F(x)}$ accordingly. Subsequently, all neighbors y∈M(x) are enumerated to update $Z_{\left\{F(x),F(y)\right\}}$ if F(x)≠F(y). Although this is the simplest and most general approach, it is not efficient for the majority of definitions of *F*. It can, however, be replaced with more efficient dedicated flooding algorithms and can be even more tuned for gradient basin definitions of *F* as we will discuss now.

### 3.1 Standard approach via global flooding

The lid method (Schön and Sibani, 1998; Sibani *et al.*, 1999) performs a ‘spreading’ enumeration starting from a local minimum with an upper energy bound for micro-states to consider, the lid. Internally, two lists are hashed: the set **D** containing all micro-states that have been processed so far and the ‘to-do-list’ **T** composed of states neighbored to **D** but not handled yet. Each processed micro-state *x* is assigned to its corresponding macro-state b=F(x) during the enumeration process. *b* is stored along with *x* in **D** and **T,** and the partition function *Z_b_* is updated by *w*(*x*) accordingly. Subsequently, all neighbors y∈M(x) of *x* with E(y)< lid-threshold are enumerated and either found in **D** or **T** (thus saving *F*(*x*) computation) or added to **T**. If the macro-state assignment for *x* and *y* differs, i.e. F(x)≠F(y), the corresponding transition state partition function $Z_{\left\{F(x),F(y)\right\}}$ is increased by $\Delta^{-1}\min(w(x),w(y))$. The method was reformulated by Kubota and Hagiya (2005) for DNA energy landscapes and Wolfinger *et al.* (2006) in the context of lattice proteins.

The barriers approach by Flamm *et al.* (2002) performs a ‘bottom-up’ evaluation of energy landscape topology based on an energy-sorted list of all micro-states in *X* above the ground state up to a predefined energy threshold. Here, the macro-state assignment *F* can be handled more efficiently compared with the lid-method, if gradient basins are applied: given that the steepest descent walk used for a gradient mapping *F* is recursive, i.e. the assignment *F*(*x*) of a state *x* is known as soon as the assignment $F(m_{\min})$ of its steepest descent neighbor $m_{\min}\in M(x)$ is known, the macro-state assignment is accomplished by a single hash lookup: because the processed set of states **D** already contains all states with energy less than *E*(*x*), looking up $m_{\min}$ and its corresponding macro-state $F(m_{\min})$ in **D** yields $F(x)\equiv F(m_{\min})$. The energy of the micro-state currently processed marks the ‘flood level’, i.e. all states in *X* with energy below have been processed. Consequently, the macro-state partition functions *Z_b_* are collected as soon as the flood level reaches the according local minimum defining *b*.

Both methods perform a massive hashing of processed states and are thus restricted by memory, i.e. the number of micro-states that can be stored in **D** and **T** is constrained to the available memory resources. Considering the exponential growth, e.g. of the RNA structure space *X* (Hofacker *et al.*, 1998), the memory is easily exhausted for relatively short sequence lengths. As the memory limit is approached, both methods result in incomplete macro-state transition data.

The barriers approach ensures a ‘global picture’ of the landscape because it covers the lower parts of all macro-states up to the reached flood level exhaustively, missing all macro-states above the limit. In case the transition states connecting the macro-states are above the flood level, no transition information is available. This can be approached by heuristics approximating the transition barrier (Bogomolov *et al.*, 2010; Flamm *et al.*, 2000a; Morgan and Higgs, 1998; Richter *et al.*, 2008; Wolfinger *et al.*, 2004); however, the outcome is still not reflecting the true targeted macro-state dynamics. In contrast, the lid method will always result in connected macro-states but only a restricted part of the landscape is covered. Furthermore, each macro-state is enumerated up to different (energy) heights resulting in varying quality of the collected partition function estimates, which further distorts the dynamics.

### 3.2 Memory-efficient local flooding

To overcome the memory limitation of global flooding approaches, we introduce a local flooding scheme. It enables parallel identification of the partition function *Z_b_* and all transition state partitions $Z_{\left\{b,c\right\}}$ for a macro-state *b* without the need of full landscape enumeration.

Similar to global flooding, the *local* approach uses a set **D** of already processed micro-states that are *part of b*, i.e. $\forall_{x\in D}:F(x)=b$, and a set **T** of micro-states that might be part of *b* or adjacent to it.

The algorithm starts in the local minimum l∈X of *b*, i.e. *F*(*l*) = *b* and $\forall_{x\neq l\in F^{-1}(b)}:E(x)>E(l)$, and does a local enumeration of micro-states in increasing energy order starting from *b*. Thus, *Z_b_* is initialized with *Z_b_* = *w*(*l*), all neighbors m∈M(l) of the minimum are pushed to **T**, and *l* is added to **D**. Afterwards, the following procedure is applied until **T** is empty.

1. Get energy minimal micro-state *x* from **T** with $\forall_{x\prime \neq x\in T}:E(x)<E(x\prime )$
2. Identify steepest descent neighbor $m_{\min}\in M(x)$ with $\forall_{m\neq m_{\min}\in M(x)}:E(m_{\min})<E(m)$
3. If $m_{\min}\in D\to F(x)=b$:
  - *x* is added to **D**,
  - *Z_b_* = *Z_b_* + *w*(*x*),
  - All neighbors m∈M(x) with *E*(*m*) > *E*(*x*) are added to **T**, and
  - Descending transitions leaving *b* are handled:
     *x* is transition state for all m∈M(x) with *E*(*m*) < *E*(*x*) and m∈D:
     $Z_{\left\{b,F(m)\right\}}=Z_{\left\{b,F(m)\right\}}+\Delta^{-1}w(x)$
    else →F(x)≠b:
  - Descending transitions entering *b* are handled:
     *x* is transition state for all m∈M(x) with *E*(*m*) < *E*(*x*) and m∈D:
     $Z_{\left\{F(x),b\right\}}=Z_{\left\{F(x),b\right\}}+\Delta^{-1}w(x)$

We use a data structure for **T** that is automatically sorted by increasing energy to boost performance of Step 1.

The algorithm computes *Z_b_* and *Z*_{_*_b_*_,_*_c_*_}_, which are required for deriving the macro-state transition rates *q_b_*_→_*_c_* [Equation (6)] from one macro-state *b* to adjacent macro-states c≠b. It is individually applied to all macro-states to get the full transition rate information of the energy landscape. Evidently, the transition state partition function *Z*_{_*_b_*_,_*_F_*_(_*_x_*_)}_, covering states between two macro-states *b* and *c*, has to be computed only once for each pair [see Equations (6 and 7)].

The major advantage of the local flooding method compared with global flooding approaches is an extremely reduced memory consumption. This is achieved by only storing the micro-states part of the current macro-state *b* (set **D**) plus all member and transition state candidates (set **T**). The reduction effect is studied in detail in the next section, and an implementation of the presented local flooding has been added to the Energy Landscape Library (ELL; Mann *et al.*, 2007). The ELL provides a generic platform for an independent implementation of algorithms and energy landscape models to be freely combined (Mann *et al.*, 2008; Mann and Klemm, 2011). Within this work, we tested our new method using the ELL-provided RNA secondary structure model, as discussed in the following section.

The reduced memory consumption of the local flooding scheme comes at the cost of increased computational efforts for the assignment of states that are not part of macro-state *b*. The above workflow does an explicit computation of *F* for all these states. Here, more sophisticated methods can be applied that either do a full or partial hashing of states partaking in steepest descent walks to increase the performance.

Another advantage is the inherent option for distributed computing, as the local flooding is performed independently for each macro-state. As such, we can yield a highly parallelized transition rate computation not possible in the global flooding scheme. This can be combined with an automatic landscape exploration approach where each local flooding instance identifies neighboring, yet unexplored, macro-states that will be automatically distributed for processing until the entire energy landscape is discovered.

We will now investigate the requirement and impact of our local flooding approach in the context of folding landscapes of RNA molecules.

## 4 FOLDING LANDSCAPES OF RNA MOLECULES

In the following, we will study the energy landscapes for the bistable RNA d33 from (Mann and Klemm, 2011) and the iron response element (IRE) of the *Homo sapiens* L-ferritin gene (GenBank ID: KC153429.1) in detail. The sequences are GGGAAUUAUUGUUCCCUGAGAGCGGUAGUUCUC and CUGUCUCUUGCUUCAACAGUGUUUGGACGGAACAG, respectively. In addition, and to evaluate the general character of our results, we generated 110 random RNA sequences with uniform base composition, 10 for each length from 25 to 35 nt. For this set average values are reported. The length restriction was a requirement for comparison with exhaustive methods.

### 4.1 Exact versus approximated transition models

We will first investigate whether exact macro-state transition probabilities are essentially required for computing a coarse-grained dynamics or whether an approximated model is providing similar results. To address this question, we performed an exhaustive enumeration of the RNA energy landscapes for d33 and IRE, resulting in ∼30 and 21 million micro-states, respectively, that are clustered into ∼2900 gradient basin macro-states for each sequence. These basins are connected by ∼60 000 macro-state transitions, representing only a fraction of 1.5% of all possible pairwise transitions.

The concept of barrier trees (Flamm *et al.*, 2002; Flamm and Hofacker, 2008) represents a straightforward approach for modeling the coarse-grained folding dynamics of an RNA molecule without explicit knowledge of the exact pairwise microscopic transition probabilities. In this context, transition probabilities between any two gradient basin macro-states *b* and *c* are defined via an Arrhenius-like equation. The latter is given in Equation (8), considering the energy difference Δ*E* between the local minimum of macro-state *b* and the lowest saddle point of any path to the target macro state *c* (which may traverse some other macro-states). The saddle point can be identified either via exhaustive enumeration (Flamm *et al.*, 2002) or estimated by path sampling techniques (Bogomolov *et al.*, 2010; Kucharík *et al.*, 2014; Li and Zhang, 2012; Lorenz *et al.*, 2009; Richter *et al.*, 2008). Energy barriers can be visualized in a tree-like hierarchical data structure, the barrier tree, resulting in all *n*^2^ pairwise transition probabilities for *n* macro-states. Coarse-grained folding kinetics based on this framework has been shown to resemble visual characteristics of the exact macro-state kinetics (Flamm *et al.*, 2002; Wolfinger *et al.*, 2004).

The Supplementary Material provides a visual comparison of coarse-grained folding dynamics for RNA d33, based on two different transition models. While the pure barrier tree dynamics resembles the overall dynamics of the two energetically lowest macro-states of the exact model well, it shows significant differences for states populated at lower extent. Given these visual discrepancies, we are interested in measuring the modeling quality of the barrier tree-based transition model versus the exact configuration. To this end, we will analyze mean first passage times (FPT) and their correlations. The FPT τ(b,t), also termed exit time (Freier *et al.*, 1986), is the expected number of steps to reach the target state t∈B from a start state b∈B for the first time (Grinstead and Snell, 1997). The first passage time for a state to itself is 0 per definition, i.e. τ(b,b)=0. For all other cases, it is defined by the recursion

(10) $$\tau (b,t)=1+\sum_{c\in B}q_{b\to c}\tau (c,t).$$

We are focused on folding kinetics, i.e. we compute the FPT from the unfolded state to all other macro-states using (i) the exact macro-state transition probabilities [Equation (6)] obsolete and (ii) the barrier tree-based transition probabilities based on the Arrhenius equation [Equation (8), barrier model].

First passage time values depend on the intrinsically unknown Arrhenius prefactor. As such, we will compare the two models using a Spearman rank correlation of the FPT, i.e. we compare the relation between FPTs rather than final values.

For d33 and IRE, the Spearman rank correlation coefficients are 0.28 and −0.12, respectively, indicating no correlation. The random sequence set shows a mean coefficient of 0.2, indicating no correlation either. No length-dependent bias was found (see Supplementary Material). Results are summarized in Table 1.

**Table 1.** Spearman rank correlation of different macro-state transition models

Sequence(s)	Spearman correlation exact–barrier	Spearman correlation exact–merged
d33	0.28	0.85
IRE	−0.12	0.64
Random	0.20	0.71

*Note*: Comparison of the Arrhenius barrier-based and the exact model shows almost no correlation, while the merged model of both is highly correlated to the exact model.

The barrier model is a simplification of the full model in two aspects: (i) *loss of precision*—the computation of transition rates based on Arrhenius-like equations is less accurate and (ii) *loss of topology*—the barrier model allows for all possible pairwise transitions, which may lead to an overestimation of transitions. To further distinguish between these two transition approaches, we have derived a merged transition model with modified transition probabilities *q^′^*. Within this merged model, $q\prime_{b\to c}$ is given by the Arrhenius-like equation [Equation (8)] for all exact macro-state transitions [$q_{b\to c}\neq 0$, Equation (6)] and zero otherwise. Investigating the Spearman rank correlation of the merged model’s FPTs with the exact FPTs, an increased correlation coefficient (0.85 for d33 and 0.64 for IRE) is observed. This is supported by a robust average coefficient of 0.71 for the set of random sequences (see Supplementary Material).

These results clearly show two key aspects of reduced folding dynamics: First, importance of the underlying topology of the landscape, i.e. the necessity to identify sparse exact transitions between macro-states, and second the reduced modeling quality when restricting the computation of transition probabilities to energy barrier-based (Arrhenius-like) approximations. The importance of the topology information for kinetics is partly studied in the Supplementary Material of Kucharík *et al.* (2014).

### 4.2 Reduction of memory requirement

Given the need for an exact computation of macro-state transition probabilities, we will now evaluate the impact of a local flooding scheme compared with the standard global flooding approach. In this context, we will investigate the memory footprint, which is the central bottleneck of global flooding methods.

As outlined above, global flooding schemes keep track of all micro-states x∈X within the energy landscape. As such, the global flooding memory consumption is dominated by mem(G)=|X|.

In contrast to that, all micro-states $x\in F^{-1}(b)$ of *b* in the local flooding scheme have to be stored to compute *Z_b_* [Equation (5)] as well as the set of all micro-state transitions leaving macro-state *b*, denoted *T*(*b*), for computing $Z_{\left\{b,*\right\}}$ [Equation (6)]. The memory consumption of local flooding of *b* is thus ruled by $\text{mem(L)}=|F^{-1}(b)|+|T(b)|$.

**Fig. 1.**
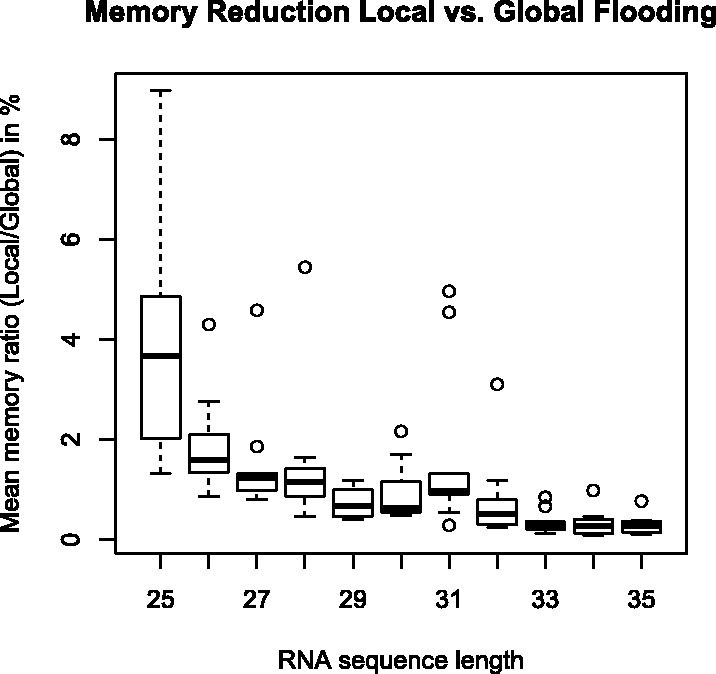
Memory consumption comparison of local versus global flooding for the random sequence set. For each RNA sequence length, 10 mean ratios of local versus global flooding memory requirement are measured and visualized in a box plot. The box covers 50% of the values and shows the median as horizontal bar. A similar picture is obtained when plotting the mean gradient basin size for each sequence

Investigating the ratio of mem(L)/mem(G) for all macro-states, we find a mean value of 0.0015 and a median of <0.0001 for both the d33 and the IRE landscape. In other words, the memory footprint of local flooding comprises <0.005 (0.5%) compared with global flooding for almost all macro-states (99%). For ∼80% of the macro-states, the footprint drops even lower to <0.01%. Similar numbers are observed within the random set for sequences of same lengths. Most notably, we see a logarithmic decrease of the average memory reduction with growing sequence length (Fig. 1). We find only three large macro-states with mem(L)/mem(G) >10% in both landscapes.

These numbers give evidence for the memory efficiency of a local flooding scheme. Within the context of extensive parallelization, such a scheme can be applied to large energy landscapes, as the individual memory consumption is several orders of magnitudes lower compared with a global flooding scheme. The remaining set of few large macro-states can be handled at the cost of longer runtimes by using the efficient local sampling scheme for macro-state transition probabilities presented in (Mann and Klemm, 2011).

### 4.3 Properties of gradient basins

In the following, we will work out various properties of gradient basins because they are commonly used as macro-state abstraction in RNA energy landscapes. We will give examples for RNA d33; however, the results can be generalized to other RNAs as shown for the random sequence set.

We have shown in the context of local flooding memory consumption that the overwhelming majority of gradient basins is small, whereas there are only a few densely populated gradient basins. Most importantly, the basin of the open, unstructured state, which is a local minimum according to the Turner energy model (Xia *et al.*, 1998) and the selected neighborhood relation *M* allows for the largest neighborhoods. Consequently, its gradient basin is the largest for all RNAs studied and wraps ∼20–30% of the state space. In the random dataset, the open state covers on average ∼40% of the landscape, and we see a decrease of this fraction with increasing sequence length. The same tendency applies to the average relative basin size (Fig. 1). Other large gradient basins are usually centered at energetically low local minima, and their basin size is in general highly specific for the underlying sequence. We do observe a correlation of basin size with the energy of its local minimum (Spearman correlation −0.73), which is in accordance to the findings of Doye *et al.* (1998) for Lennard–Jones clusters.

**Fig. 2.**
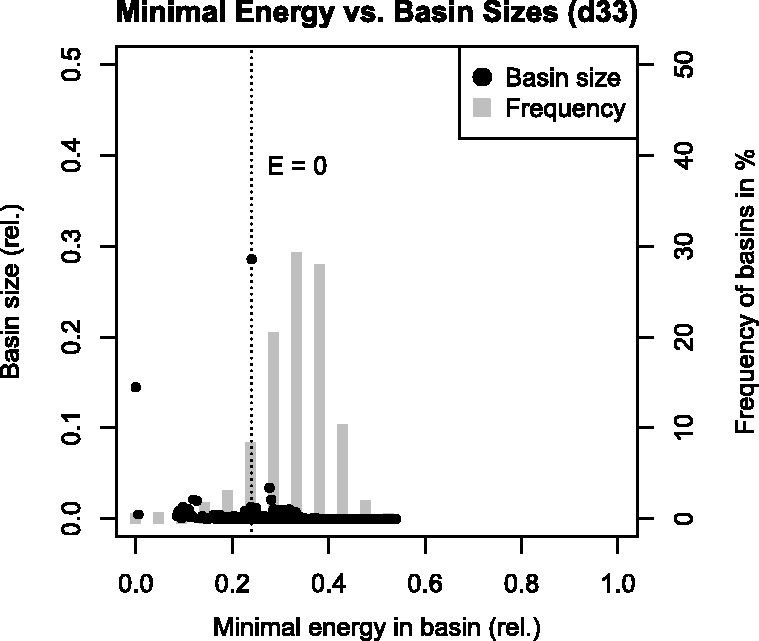
Distribution of basin sizes (dots) and frequency histogram of basins (bars) over the energy range within the energy landscape of RNA d33. Relative energies are given by $E_{\text{rel}}(x)=(E(x)-E_{\min})/(E_{\max}-E_{\min})$ where $E_{\min}/E_{\max}$ denote the energy boundaries over *X*. The dotted line marks the position of the unstructured state with energy 0

When investigating the distribution of the energetically lowest micro-states in each gradient basin, i.e. the local minima, we find that most minima have positive energies (see histogram in Fig. 2). Minima are distributed over the lower 40–50% of the energy range for all sequences studied. The number of minima with negative energy, i.e. stable secondary structures, is ∼100 for d33 and IRE and is in the range of ∼5% in general for the random set studied here. The majority of the state space of RNA energy landscapes shows positive energies, resulting from destabilizing energy terms for unstacked base pairs in the Turner energy model (Xia *et al.*, 1998). This is in accordance with the results from Cupal *et al.* (1997) who found that only ∼10^6^ of ∼10^16^ structures of a tRNA show an energy of less than zero.

**Fig. 3.**
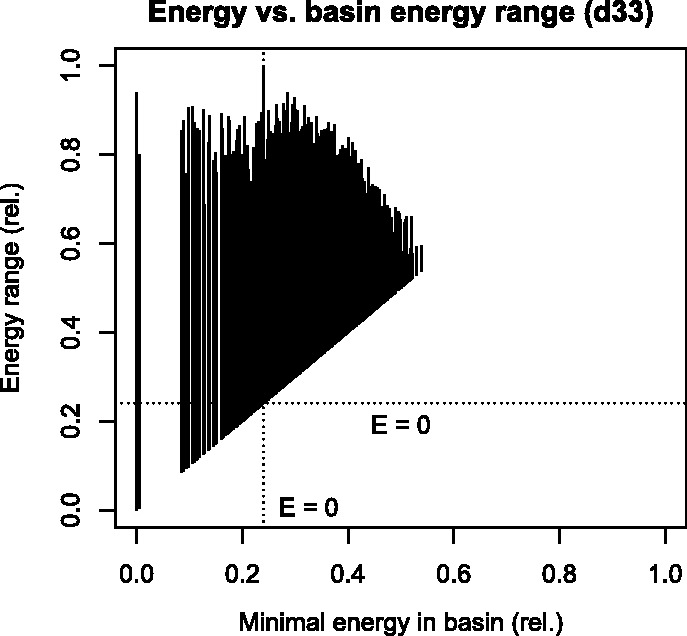
The energy range covered by each basin (*Y*-axis) sorted by the minimal energy within the basin (*X*-axis) over the whole energy range of the energy landscape of RNA d33. Relative energies are given by $E_{\text{rel}}(x)=(E(x)-E_{\min})/(E_{\max}-E_{\min})$ where $E_{\min}/E_{\max}$ denote the energy boundaries over *X*. The dotted lines mark the position of the unstructured state with energy 0

The energy range of most gradient basins, i.e. minimal to maximal energy of any micro-state in the basin as plotted in Figure 3, covers almost the entire range above a local minimum. This is generally independent of the basin size (compare Fig. 2 and 3); only for energetically high basins a lower maximal energy is observed. This might be a result of the accompanying basin size decrease or an artifact of the energy model. The gradient basin of the unstructured state covers the energetically highest states.

As mentioned above, only few of the possible |*B*|^2^ macro-state transitions are observed. We find that >50% of the basins show <10 neighboring basins and almost all (98%) have transitions to <2% of the basins. The gradient basin of the unstructured state shows the highest number of macro-state transitions and is connected to >20% of the macro-states. We find that few large basins serve as hub nodes with high connectivity. This is in accordance to findings of Doye (2002) for Lennard–Jones polymers. Consequently, the number of transitions is highly correlated to the basin size, as one would expect. This is supported by a Spearman rank correlation coefficient of ∼ 0.8 for all RNAs studied. The correlation to the basin’s minimal energy, as found by Doye (2002), is not as significant (Spearman correlation −0.6).

## 5 CONCLUSION

We have introduced a local flooding scheme for computing the exact macro-state transition rates for arbitrary discrete energy landscapes, provided some macro-state assignment is available. The approach has been evaluated on RNA secondary structure energy landscapes in the context of modeling coarse-grained RNA folding kinetics based on gradient basins. We have demonstrated the need for exact macro-state transition models via comparison with a simpler barrier tree-based Arrhenius-like model. The latter resulted in significantly different dynamics measured by mean FPT.

We showed that the local flooding scheme requires several orders of magnitude less memory compared with the standard global flooding scheme. Furthermore, it is intrinsically open to vast parallelization, which should also result in significant runtime reduction, given that the global flooding can not be easily parallelized.

Finally, we performed a thorough investigation of gradient basins in RNA energy landscapes because they are commonly used as macro-state abstraction in the field. Gradient basins have been shown to be generally small, which is the reason for the tremendously reduced memory requirement of the local flooding scheme. The basin of the unstructured state has been shown to be special, as it is the largest, most connected macro-state and covers the energetically highest micro-states. Independent of their size, most basins contain micro-states of almost the entire energy range above their respective local minimum. The majority of the gradient basins covers only states with positive energy. We found a low average connectivity between gradient basins, the existence of few highly connected hub nodes and a high correlation of connectivity with basin size.

*Funding*: This work was partly funded by the Austrian Science Fund (FWF) project ‘RNA regulation of the transcriptome’ (F43), the EU-FET grant RiboNets 323987, the COST Action CM1304 ‘Emergence and Evolution of Complex Chemical Systems’ and by the IK Computational Science funded by the University of Vienna.

*Conflicts of Interest*: none declared.

## Supplementary Material

Supplementary Data
