# Supplementary material for: Memory-efficient RNA energy landscape exploration
Source: Bioinformatics. 2014 May 14;30(18):2584–91. doi: 10.1093/bioinformatics/btu337 (PMC4155248; doi:10.1093/bioinformatics/btu337)
Supplement: Supplementary Data [file supp_30_18_2584__index.html]

Memory efficient RNA energy landscape exploration — Memory-efficient RNA energy landscape exploration — Memory-efficient RNA energy landscape exploration — Supplementary Data 

# Memory-efficient RNA energy landscape exploration

## Supplementary Data

files

**Files in this Data Supplement:**

- Supplementary Data - pdf file
