# Supplementary material for: Memory-efficient RNA energy landscape exploration
Source: Bioinformatics. 2014 May 14;30(18):2584–91. doi: 10.1093/bioinformatics/btu337 (PMC4155248; doi:10.1093/bioinformatics/btu337)
Supplement: Supplementary Data [file supp_btu337_el-explore-suppl.pdf]

Memory efficient RNA energy landscape exploration
# - Supplementary Material -

Martin Mann [1,*], Marcel Kucharík [2], Christoph Flamm [2] and Michael T. Wolfinger [2,3,4]

[1] Bioinformatics Group, University of Freiburg, Georges-Köhler-Allee 106, D-79110 Freiburg, Germany
[2] Institute for Theoretical Chemistry, University of Vienna, Währingerstraße 17, 1090 Vienna, Austria
[3] Center for Integrative Bioinformatics Vienna (CIBIV), Max F. Perutz Laboratories, University of Vienna & Faculty of Computer Science, University of Vienna, Dr. Bohr-Gasse 9, 1030 Vienna, Austria.
[4] Department of Biochemistry and Molecular Cell Biology, Max F. Perutz Laboratories, University of Vienna, Dr. Bohr-Gasse 9, 1030 Vienna, Austria

1 Exact vs. approximated transition models

Figure 1 presents the Spearman rank correlation of the mean first passage times (FPT) for the different transition probability models studied. The plot is based on the random data set and grouped by sequence length.

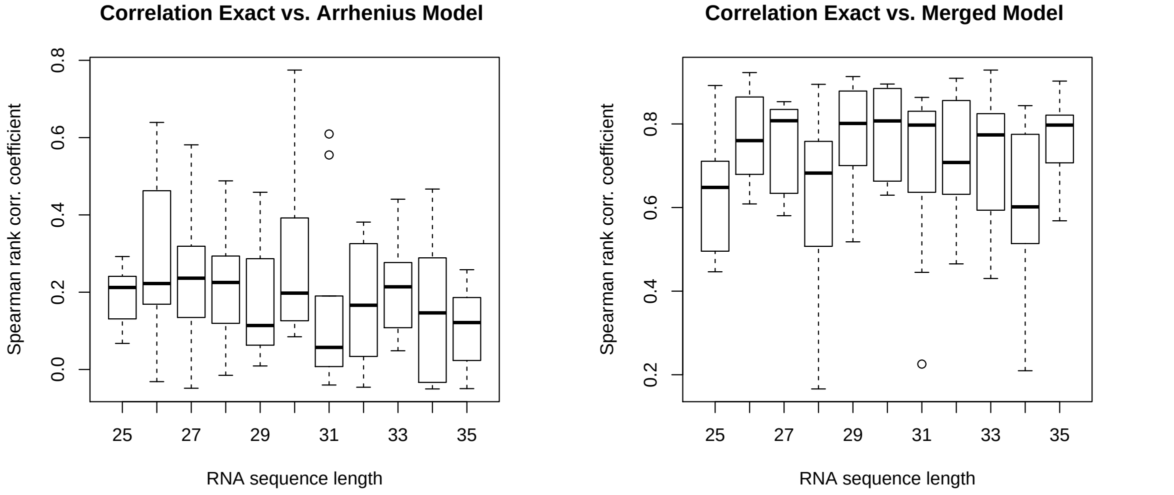

Figure 1: Spearman rank correlation coefficients of the mean first passage times (FPT) for the random data set grouped by sequence length. Correlation of the exact model (left) with the Arrhenius barrier-based transition model (right) and the merged transition probability model.

*to whom correspondence should be addressed: `http://www.bioinf.uni-freiburg.de`

Figure 2 provides a visual comparison of coarse-grained folding dynamics for RNA d33, based on two different transition models. While the pure barrier tree dynamics (lower plot) resembles the overall dynamics of the two energetically lowest macro-states of the exact model (upper plot) quite well, it shows significant differences for states populated at lower extent (*e.g.* at rank 5 or 6).

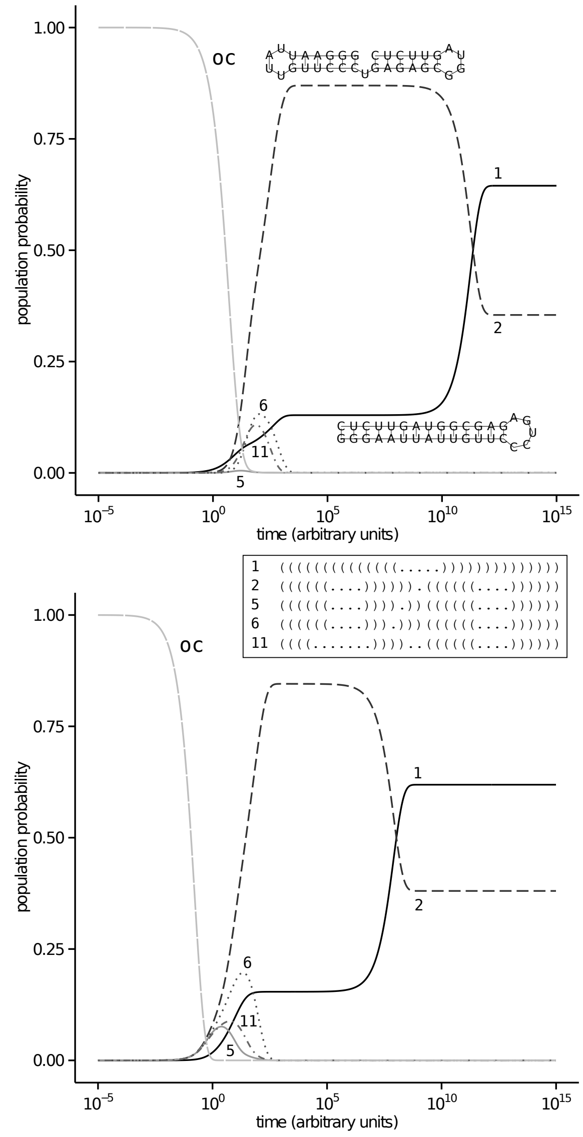

Figure 2: Coarse-grained folding dynamics of RNA d33 showing the five most populated gradient basins. Each curve represents the population probability of a gradient basin macro state, depicted by the secondary structure of its local minimum. Numbers correspond to energy sorted ranks. Simulations were started from the unstructured open chain macro-state (`oc` curve) and let evolve until a stationary distribution of the underlying Markov process was reached, see Wolfinger *et al.* (2004) for details. We compare the dynamics from exact transition probabilities (left) to those from a barrier tree-based Arrhenius transition model (right).

2 Memory Consumption Local vs. Global Flooding

In Figure 3 on the left, we present the memory consumption of the local vs. the global flooding approach in terms of number of structures to be kept in memory for the random RNA sequence set. The local flooding requires several orders of magnitude less memory compared to global flooding. As expected, a growth in sequence length is visible.

The right side of Figure 3 presents the distribution of gradient basin sizes over the energy range for RNA d33. A decrease in basin size is observed with increasing minimal energy. A similar result was found in the context Lennard-Jones clusters by Doye *et al.* (1998).

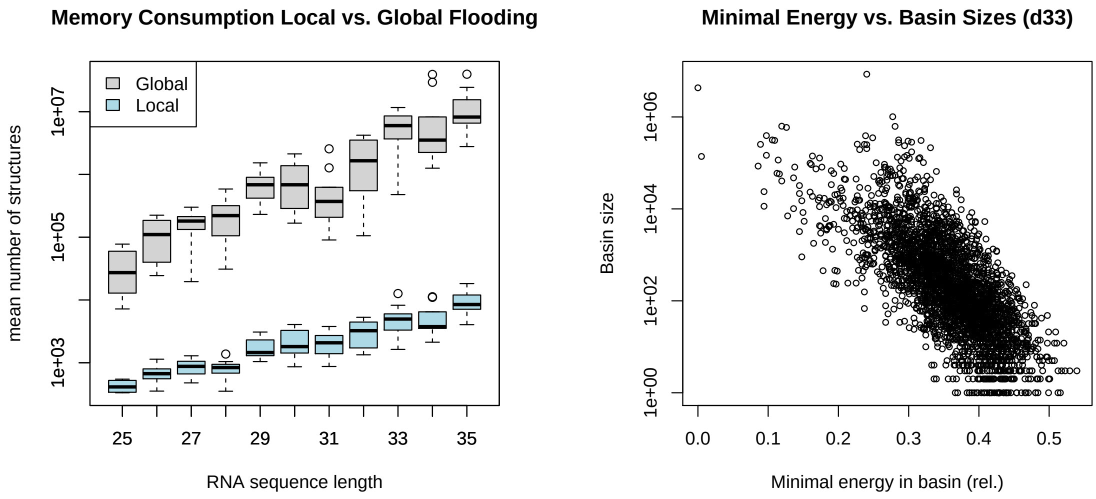

Figure 3: Memory consumption of global and local flooding for different RNA lengths within the random data set (left). Distribution of gradient basin sizes on a logarithmic scale over the energy range for RNA d33 (right).

References

Doye, J. P. K., Wales, D. J., and Miller, M. A. (1998). Thermodynamics and the global optimization of Lennard-Jones clusters. *The Journal of Chemical Physics*, **109**(19), 8143–8153.

Wolfinger, M. T., Svrcek-Seiler, W. A., Flamm, C., Hofacker, I. L., and Stadler, P. F. (2004). Efficient computation of RNA folding dynamics. *J. Phys. A: Math. Gen.*, **37**, 4731–4741.
